# Singlet–triplet annihilation in single LHCII complexes†

**DOI:** 10.1039/c5cp01806d

**Published:** 2015-07-03

**Authors:** J. Michael Gruber, Jevgenij Chmeliov, Tjaart P. J. Krüger, Leonas Valkunas, Rienk van Grondelle

**Affiliations:** a Department of Biophysics, Faculty of Sciences, Vrije Universiteit De Boeleaan 1081 1081HV Amsterdam The Netherlands j.m.gruber@vu.nl r.van.grondelle@vu.nl; b Department of Theoretical Physics, Faculty of Physics, Vilnius University Saulėtekio Ave. 9 LT-10222 Vilnius Lithuania; c Institute of Physics, Center for Physical Sciences and Technology Goštauto 11 LT-01108 Vilnius Lithuania; d Department of Physics, Faculty of Natural and Agricultural Sciences, University of Pretoria Private bag X20 Hatfield 0028 South Africa

## Abstract

In light harvesting complex II (LHCII) of higher plants and green algae, carotenoids (Cars) have an important function to quench chlorophyll (Chl) triplet states and therefore avoid the production of harmful singlet oxygen. The resulting Car triplet states lead to a non-linear self-quenching mechanism called singlet–triplet (S–T) annihilation that strongly depends on the excitation density. In this work we investigated the fluorescence decay kinetics of single immobilized LHCIIs at room temperature and found a two-exponential decay with a slow (3.5 ns) and a fast (35 ps) component. The relative amplitude fraction of the fast component increases with increasing excitation intensity, and the resulting decrease in the fluorescence quantum yield suggests annihilation effects. Modulation of the excitation pattern by means of an acousto-optic modulator (AOM) furthermore allowed us to resolve the time-dependent accumulation and decay rate (∼7 μs) of the quenching species. Inspired by singlet–singlet (S–S) annihilation studies, we developed a stochastic model and then successfully applied it to describe and explain all the experimentally observed steady-state and time-dependent kinetics. That allowed us to distinctively identify the quenching mechanism as S–T annihilation. Quantitative fitting resulted in a conclusive set of parameters validating our interpretation of the experimental results. The obtained stochastic model can be generalized to describe S–T annihilation in small molecular aggregates where the equilibration time of excitations is much faster than the annihilation-free singlet excited state lifetime.

## Abstract 2

The two-exponential fluorescence decay kinetics of single LHCII complexes are quantitatively explained by a stochastic model of singlet–triplet annihilation.

## Graphical abstract

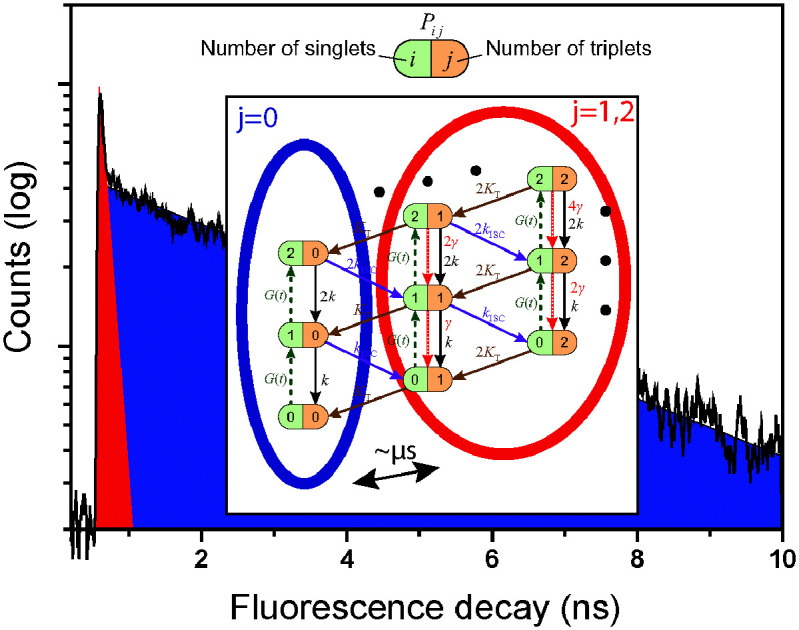

## 1 Introduction

Solar radiation is the most abundant source of energy on earth. Over billions of years of evolution some organisms have learned how to utilize and then store it in the form of chemical energy needed for their vital activity. This process, called photosynthesis, turned out to be extremely important to sustain life on our planet by providing a primary source of biomass as well as saturating Earth's atmosphere with oxygen, a byproduct of photosynthesis required for the vast majority of heterotrophic organisms. The two photosystems of green plants and algae—photosystem I and photosystem II (PSII)—operate in series and are composed of large ensembles of chlorophyll (Chl) and carotenoid (Car) molecules bound to the protein scaffold and distributed over the thylakoid membrane.^1^ The spectroscopic properties and mutual arrangement of these pigments within the light-harvesting antenna of photosystems ensure optimal absorption of the incoming electromagnetic radiation followed by highly efficient delivery of the generated electronic excitations to a reaction center (RC).^1,2^ Subsequent charge separation in the RC is the initial step of a series of trans-membrane electron and proton transfer events that convert the electronic excitation to chemical energy.^3^

The major photosynthetic light-harvesting complex (LHCII) is the main antenna complex of PSII and binds over 50% of all terrestrial thylakoid Chls.^4–6^ The high-resolution crystal structure of LHCII reveals its trimeric nature, with each monomeric subunit containing eight Chls *a*, six Chls *b*, and four Cars (2 luteins, neoxanthin, and violaxanthin or zeaxanthin).^7^ Cars not only increase the total absorption cross-section by utilizing green light not accessible for Chls, but also play an important photoprotective role.^8^ In low light conditions, almost all generated excitons are successfully delivered to the RC and then used for charge separation. However, due to a finite turnover rate of the RCs, intense illumination can lead to over-excitation of the light-harvesting antennae. In such conditions, charge recombination in the RC and triplet formation in the light harvesting antennae result in a quantum yield of Chl triplet generation of about 30%.^9^ The resulting Chl triplet states decay on a millisecond timescale^10,11^ and therefore readily react with molecular oxygen to form singlet oxygen, which is highly reactive and therefore toxic to proteins and lipids.^12^ Cars are known to successfully scavenge this reactive oxygen species and dissipate its energy as heat.^13^ Moreover, it was found that in LHCII more than 90% of Chl triplets are at room temperature efficiently quenched primarily by two lutein molecules thus even avoiding the formation of singlet oxygen.^14,15^

The extensive studies of the excitation energy transfer within LHCII show very fast Chl *b* to Chl *a* relaxation, occurring on a timescale of several ps.^4,16–19^ Due to the much slower total singlet excited state decay of LHCII (lifetime of isolated LHCII ≈ 3.5 ns) and an inter-system crossing rate of ∼10 ns^−1^, mainly Chl *a* triplet states are formed.^14,20^ From the crystal structure it can be seen that all Chls *a* are in close proximity with either one of two central luteins or neoxanthin.^7,21^ This spatial arrangement of the pigment molecules leads to efficient quenching of the Chl triplet states.^22^ The fourth Car, either violaxanthin or zeaxanthin depending on the stress conditions of plants or algae before protein purification, is located at the periphery of the protein backbone and was shown not to contribute to triplet quenching.^23,24^

The resulting triplet states of Car molecules can also act as an intrinsic photo-protection mechanism, which under high photon flux conditions quenches singlet excited states of Chls *via* non-linear exciton–exciton annihilation.^25^ The efficiency of this S–T annihilation process depends on the excitation intensity, the exciton diffusion radius, the number of pigments within the system, and their connectivity.^26–29^

While investigating the fluorescence from photosynthetic complexes, much effort is usually required to achieve annihilation-free conditions thus simplifying modeling approaches and the interpretation of the obtained results. However, in some ensemble measurements and especially in single-molecule experiments the excitation intensities are often so high that annihilation cannot be avoided. Recently it has been shown that singlet–triplet (S–T) annihilation can have a significant effect on extended conjugated polymer structures, where this kind of self-quenching results in a decreased fluorescence yield.^30^ As a result, this photo-physical process can also ultimately lower the overall yield of free charge carriers in organic solar cell applications, where long-range energy transfer sometimes cannot be avoided.

The annihilation kinetics in molecular aggregates are usually diffusion-limited and well-described with a rather simple kinetic model.^31^ This kinetic approach has been successfully applied to aggregates of LHCIIs.^32,33^ It has also been used to describe the saturation of the steady-state fluorescence with increasing excitation intensity of single LHCII complexes.^34^ However, this kinetic model did not give correct solutions for the time-resolved fluorescence decay kinetics of LHCII trimers. Meanwhile, it was demonstrated that non-linear singlet–singlet (S–S) annihilation kinetics in LHCII trimers can be reproduced well by a stochastic model.^32,33^

In this work we investigate the fluorescence kinetics of single LHCII trimers^35^ by means of single molecule spectroscopy (SMS) and focus on the observed excitation intensity-dependent kinetics of fluorescence quenching. The SMS approach enables us to exclude statically quenched and photo-bleached complexes which is, especially at the necessary high excitation intensities, a big advantage over ensemble measurements. The observed two-exponential fluorescence decay kinetics and time-dependent changes in the fluorescence intensity, recorded in the microsecond time range, exhibit features indicative of S–T annihilation.^36^ To verify this conclusion, a stochastic model for S–T annihilation is developed and successfully applied to quantitatively describe all the experimental observations.

## 2 Materials and methods

### Sample preparation

LHCII complexes in their trimeric form were isolated from spinach thylakoids, as described previously.^37^ During the last step the sample was purified *via* fast protein liquid chromatography (FPLC) in order to reduce the content of monomeric LHCIIs and free pigments and then frozen only once. The thawed sample was diluted down to a concentration of ∼10 pM in a measuring buffer (25 mM Hepes, pH 7.5 and 0.03% (w/v) *n*-dodecyl β-d-maltoside) and then immobilized on a PLL (poly-l-lysine, Sigma Aldrich) coated cover glass. The final concentration was determined empirically to achieve a density of surface bound complexes of roughly 10 complexes per 100 μm^2^.^38^ The closed sample chamber with a volume of ∼100 μl also contained an oxygen scavenging system of 2.5 mM protocatechuic acid and 25 nM protocatechuate-3,4-dioxygenase (Sigma Aldrich) to reduce photobleaching and enhance photostability of the complexes.^39^

### Single molecule spectroscopy and data analysis

A confocal microscope was used to investigate the fluorescenceproperties of single complexes at room temperature, as described earlier.^38,40^ The sample was excited at 633 nm utilizing a Ti:sapphire laser (Coherent MIRA 900F) with a pulse width of 200 fs and a repetition rate of 76 MHz, coupled to a tunable optical parametric oscillator (Coherent MIRA OPO). Near-circular polarized light was obtained by means of a Berek polarization compensator (5540 New Focus). Before measuring the fluorescence kinetics of single complexes, a fluorescence spectrum with one-second integration time was obtained for each complex by dispersing the fluorescence light *via* a grating (Optometrics LLC, HR830/800 nm) onto a CCD camera (Roper Scientific, Spec10:100BR). That allowed us to identify and exclude any spectrally shifted and denatured photosynthetic complexes from the subsequent analysis.^38^ The wavelength-integrated fluorescence was measured with a single photon avalanche diode (Micro Photon Devices, PDM series, diameter of active area: 20 μm). A time-correlated single photon counting (TCSPC) device (PicoHarp 300, PicoQuant) allowed us to acquire both the absolute and relative (triggered by pulsed laser excitation) arrival time of the detected photons. The absolute photon arrival times were used to generate 10 ms binned fluorescence intensity traces which were analyzed with a self-written Matlab code to identify intensity levels, as described earlier.^41^ Only unquenched states were analyzed and blinking events were excluded. A typical time trace (black line) and the fitted intensity levels (red line) are illustrated on the left side of Fig. S1 in the ESI.† The corresponding relative arrival times of detected photons within only one intensity level were binned into 4 ps time intervals, and the resulting histogram (right side of Fig. S1 in the ESI†) was first corrected by subtracting a measured and time-weighted background signal and then further analyzed with the software FluoFit (PicoQuant). A dichroic mirror (Z633RDC, Chroma Technology Corp.) and a fluorescence filter (HQ645LP, Chroma Technology Corp.) filtered out most of the excitation light, but a small fraction of leaking laser light was subtracted *via* background correction. Control experiments were done with additional long pass fluorescence filters to completely suppress the leakage of laser light. The fluorescence lifetimes were obtained by an exponential reconvolution fit using an instrument response function (IRF) measured from scattered light at the peak emission wavelength of LHCII (*λ* = 681 nm). The IRF at the excitation wavelength of 633 was measured as a control and was found to be identical. The full width at half maximum (FWHM) of the IRF was 38 ps for both wavelengths and is dominated by the timing error of the detector. The quality of the fitting procedure was evaluated from the lack of structure in the fit residuals and their auto-correlation function. The high repetition rate of the laser limits the time range of the fluorescence decay to 13.16 ns and results in an incomplete decay. However, the time constants of a multi-exponential decay are not affected, and the error associated with the relative amplitude of the slowest decay component in our measurements is less than 3% and is furthermore taken into account within the fitting software.^42^

To measure time-dependent fluorescence intensity changes in the microsecond range, the excitation was periodically modulated by utilizing an acousto-optic modulator (MT350, Acousto-Optic Devices), as shown in the inset of Fig. 3. By setting the frequency, 1/(*t*_on_ + *t*_off_), and duty cycle, *t*_on_/(*t*_on_ + *t*_off_), of a periodical step function that determines the amplitude of transmitted excitation light, one can essentially use the AOM as a fast shutter with adjustable on- and off-times (see inset of Fig. 3 for notation). The absolute photon arrival times can be projected back in one modulation cycle, which allows to build up a photon histogram (AOM histogram) that describes the fluorescence intensity kinetics within the on-time of one modulation cycle. Slow envelope drifts of the absolute arrival time due to the TCSPC electronics were corrected *via* subtracting a moving average function.

## 3 Experimental results

The fluorescence decay of a single LHCII complex at excitation intensities *I*_E_ ≲ 50 W cm^−2^ can be satisfyingly fitted (deconvoluted) with a single-exponential function: *F*(*t*) ∝ exp(−*t*/*τ*_slow_). The obtained mean value of the excitation lifetime, *τ*_slow_ = (3.4 ± 0.3) ns, which was measured individually in about 100 single unquenched complexes, is the same as the mean fluorescence lifetime in an ensemble of solubilized complexes, *τ* = (3.45 ± 0.02) ns, measured on the same setup (Fig. S2 in the ESI†). However, when the excitation intensity is increased, two decay components are necessary to reproduce the excitation kinetics, so that

**Fig. 1:**
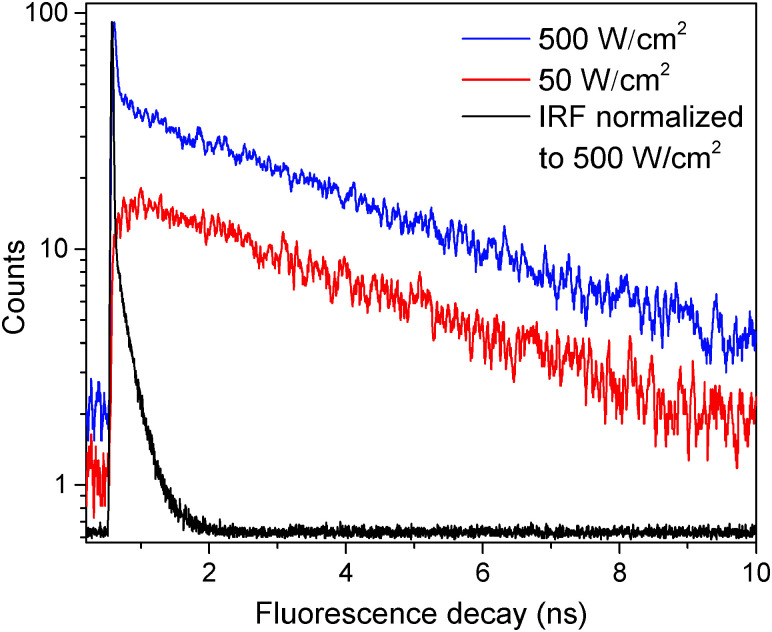
Fluorescence decay traces of a single LHCII complex at different excitation intensities of 50 (red line) and 500 W cm^−2^ (blue line). The black line indicates the instrument response function (IRF) with a full width at half-maximum of 38 ps.

**Fig. 2:**
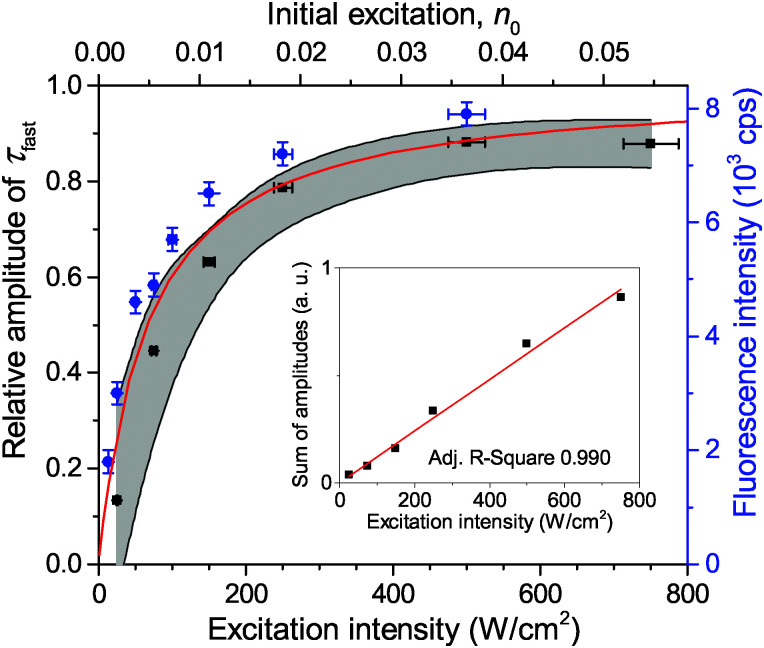
Dependence of the relative amplitude of a fast 35 ps component on the excitation intensity. The black squares show the experimentally measured mean values of about 20 LHCII complexes and the gray shading illustrates the corresponding standard deviation. The fluorescence intensity saturation behavior of a single LHCII complex is depicted with blue squares (right axis). The red line is the theoretically calculated amplitude ratio depending on the initial number of excitations per pulse, *n*_0_ (top axis). The inset shows the linear correlation between the total amplitude, *A*_fast_ + *A*_slow_, and the excitation intensity.

1 *F*(*t*) = *A*_slow_e^−*t*/*τ*_slow_^ + *A*_fast_e^−*t*/*τ*_fast_^,

where *A*_slow_ and *A*_fast_ denote the amplitudes of the slow and fast lifetime component, respectively. As an example, Fig. 1 presents two fluorescence decay traces on a semi-logarithmic scale and clearly demonstrates the appearance of a second fast decay component *τ*_fast_ at the higher excitation intensity of 500 W cm^−2^. Moreover, the slow component *τ*_slow_ turned out to be independent of the excitation intensity. However, the overall fluorescence intensity did increase with the excitation intensity, followed by a saturation behavior at intensities *I*_E_ ≳ 500 W cm^−2^, as demonstrated with blue squares in Fig. 2.

In order to further investigate the nature of the fast component, the fluorescence kinetics were measured at an excitation intensity of 750 W cm^−2^ and then fitted according to eqn (1), which resulted in a value of *τ*_fast_ = 35 ± 10 ps. A whole histogram of the fitted lifetimes of 100 individually measured and analyzed LHCII trimers is presented in Fig. S3 in the ESI.† Interestingly, the obtained value of the fast decay component *τ*_fast_ lies within the time range of less than 40 ps, reported for annihilation processes, and within the limits of slow energy transfer processes (equilibration time) in an LHCII trimer.^32,43–45^ This result, together with the previously observed dependence of the fluorescence intensity on the excitation power,^34^ suggests that the fast decay component seen in our experiments in principle might be connected to S–T annihilation, though the current model for S–T annihilation in molecular aggregates^31^ cannot explain the appearance of excitation intensity-dependent two-exponential decay kinetics.

Measurements of *τ*_fast_ at a lower excitation intensity of 300 W cm^−2^ yielded the same result. Therefore we assumed a fixed value of *τ*_fast_ = 35 ps for further analysis thus preventing any possible misfitting of *τ*_fast_ at excitation intensities below 300 W cm^−2^. In order to quantify the contribution of *τ*_fast_ to the overall decay kinetics, the relative amplitude of the fast decay component, *A*_fast_/(*A*_fast_ + *A*_slow_), was calculated. The mean dependence of this relative amplitude on the excitation intensity, obtained from a set of about 20 single LHCII trimers, is shown with black squares in Fig. 2. Meanwhile, the sum of both amplitudes (*A*_fast_ + *A*_slow_) of non-normalized fluorescence kinetics in a single complex correlates with the excitation intensity, as shown in the inset of Fig. 2. This is a complimentary check for the fitting procedure because the sum of amplitudes is proportional to the initial number of excitations generated per trimer and should therefore scale linearly with the excitation intensity. The obtained results thus validate our assumption of the fixed lifetime *τ*_fas*t*_ and exclude the presence of any additional, possibly unresolved fast decay component.

**Fig. 3:**
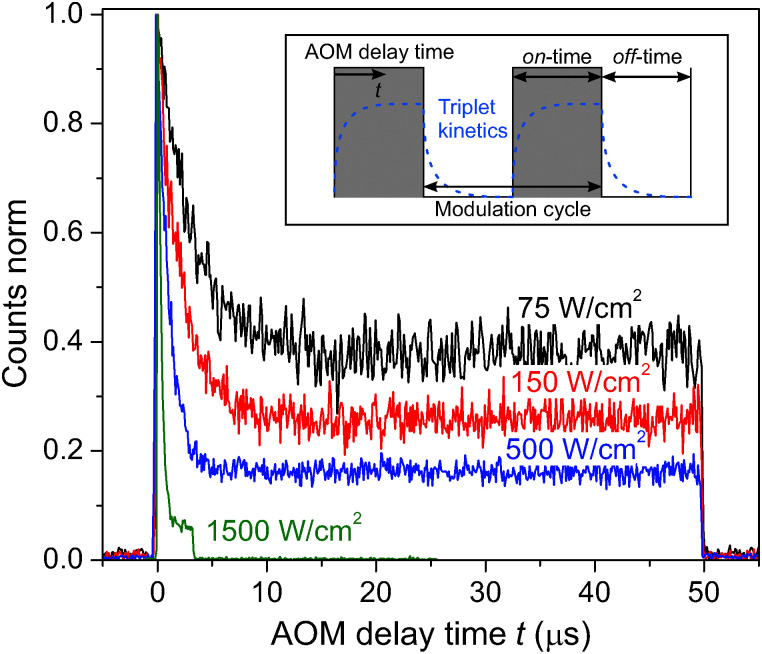
Fluorescence intensity kinetics of single LHCII complexes after the onset of illumination (at *t* = 0) for different excitation intensities and the same excitation modulation, *t*_on_ + *t*_off_ = 50 μs + 50 μs = 100 μs. These kinetics (AOM histograms) were extracted by histogramming the absolute fluorescence photon arrival times into one modulation cycle with a binning time of 100 ns (gray-shaded region in the inset). The measurement at the highest excitation intensity (green curve) had a shorter on-time of 3.3 μs in order to prevent fast photo-bleaching at such a high excitation intensity. Inset: Illustration of the stepwise amplitude modulation of the excitation light *via* an acousto-optic modulator. By effectively turning the excitation laser on and off on the μs time range we can control the time-dependent changes in the concentration of triplets, as schematically shown with the dashed blue line. The varying concentration of triplets can then be observed *via* the measured fluorescence intensity kinetics.

So far only the steady state conditions of the involved fluorescence decay kinetics, detected during a continuous measurement on a time scale of several seconds, have been discussed. By utilizing an acousto-optic modulator (AOM) as a fast shutter we can perform conditional measurements and study the time-dependent decay kinetics in the micro-second time range. The inset in Fig. 3 illustrates a typical stepwise binary modulation of the excitation laser power. It allows us to test the hypothesis whether triplet states, governing S–T annihilation, are correlated to the 35 ps decay component. If so, during the illumination period the population of triplet states in the system should increase with the AOM delay time *t*, while it should drop when the illumination is switched off, as schematically shown in the inset of Fig. 3. Cars are known to very efficiently quench Chl triplet state, thus, if our assumption is correct, eventually Car triplets are generated. The reported time scale of the Car triplet decay, *K*_T_^−1^, varies between 2–4 μs for aerobic and 7–9 μs for anaerobic conditions.^14,46^ As a result, notable variations of the Car triplet population should be expected in the μs time range, and S–T annihilation should lead to strong variations in the fluorescence intensity. By using the time-tagged absolute arrival time of a detected fluorescence photon, the detection events can be histogrammed into the time interval of a single modulation cycle (gray-shaded area in the inset of Fig. 3). The resulting kinetics, shown in Fig. 3, indeed illustrate the time dependent decrease of fluorescence that can be attributed to the increasing cumulative probability of triplet state formation during the on-time of the modulation cycle, thus supporting our assumption on the dominating role of S–T annihilation. The on-time for the highest excitation intensity was shortened to 3.3 μs in order to avoid fast photobleaching of the complex while it still reached steady-state conditions (plateau). There is a peak of fluorescence intensity at the onset of excitation because the probability to have a triplet state in the system decreased during the preceding AOM off-time of *t*_off_ ≧ 50 μs to below 1%. The time constant and the amplitude offset of the normalized kinetics notably drop with increasing excitation intensity. The reason for such a behavior is more pronounced formation of triplet states, resulting in a higher probability for S–T annihilation events with singlet states of Chl molecules. The final steady-state population of triplets upon increasing the excitation density during the illumination period is then increasing as well, thus lowering the steady-state fluorescence signal. This can further be illustrated by plotting the fluorescence decay at different AOM delay times, as shown in in Fig. S4 in the ESI.† At the onset of illumination there is no fast lifetime component, corresponding to the overall singlet excited state decay without annihilation. Later on the fast component is dominating the fluorescence decay. This time-dependent accumulation proves that the fast decay component is not an artifact.

**Fig. 4:**
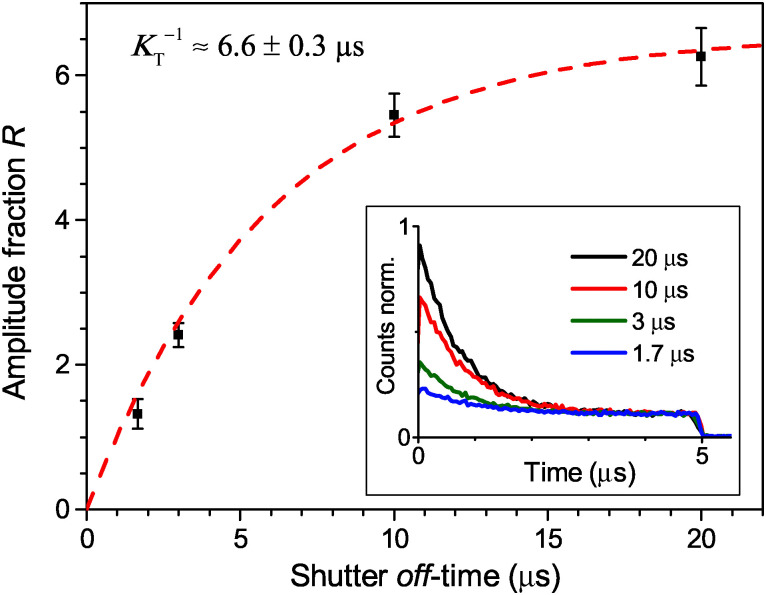
Direct measurement of the Car triplet decay rate *K*_T_ by stepwise modulation of the excitation intensity with altering shutter off-time *t*_off_. The relative fluorescence amplitude at the onset of an excitation modulation cycle increases asymptotically from the steady state value of *R* = 0 under continuous illumination (for *t*_off_ = 0) to an approximately annihilation-free plateau for sufficiently long off-times (*t*_off_ > 20 μs). The black squares are the mean values, the error bars illustrate the standard deviation of 5 LHCII complexes per single *t*_off_ value, and the red line indicates a single-exponential fit according to eqn (3). The inset shows the corresponding fluorescence histograms measured with different *t*_off_ at an excitation intensity of 500 W cm^−2^.

Finally, the lifetime of the generated triplet state can also be evaluated by modulating the excitation intensity. Indeed, variations in the off-time period, *t*_off_, of the AOM shutter in the time range of *K*_T_^−1^ indirectly probe the exponential decay of the triplet state population. By choosing the on-time of the AOM shutter as 5 μs at an excitation intensity of 500 W cm^−2^, the triplet population reaches approximately steady state conditions during the on-time of the AOM shutter. The fluorescence histograms, measured for the same excitation intensity, the same *t*_on_ = 5 μs and four different *t*_off_ values, are shown in the inset of Fig. 4. These histograms, normalized at their steady-state amplitudes *A*_st_, corresponding to the steady-state triplet concentration, can be readily fitted with a single-exponential function of the form

2 *F*_AOM_(*t*) = *A*_st_ + *A*_0_ exp(−*t*/*τ*_AOM_),

with *τ*_AOM_ ≈ 0.8 μs. The amplitude *A*_0_ at the onset of a modulation cycle, normalized to the steady-state amplitude *A*_st_, reflects the decrease in the Car triplet state population for a certain off-time period *t*_off_. The extracted relative amplitude ratios *R*(*t*_OFF_) = *A*_0_(*t*_OFF_)/*A*_st_ are depicted with black squares in Fig. 4, and the red line shows the fitted exponential fluorescence recovery,

3 *F*_Recovery_(*t*) = *A*[1 − exp(−*K*_*T*_·*t*)],

with a time constant of *K*_T_^−1^ ≈ 6.6 μs. As expected, this value perfectly lies within the mentioned range of 2–9 μs, reported for the lifetimes of Car triplet states.^14,46^

## 4 Modeling

The experimental results provided in the previous section describe the observed kinetics in a qualitative way in terms of S–T annihilation. In order to obtain quantitative values for the underlying decay rates and to further validate and explain the experimental results, we need to test them with an appropriate model. In previous studies of S–T annihilation in large aggregates of chromophores, the excitation kinetics were usually well described by a simple kinetic model:^31^4
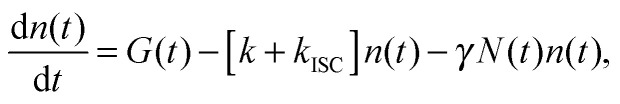
5
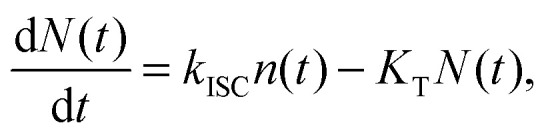
where *n*(*t*) and *N*(*t*) denote the time-dependent concentrations of singlets and triplets, respectively; *k* and *K*_T_ are the rate constants of the linear singlet and triplet exciton decay, respectively; *γ* is the rate constant of S–T annihilation; *k*_ISC_ is the rate of inter-system crossing in a chromophore molecule; and *G*(*t*) is the singlet generation rate (pumping rate). Since *k* and *K*_T_ usually differ by several orders of magnitude, the change in triplet concentration in the steady-state regime between two subsequent laser pulses is almost negligible compared to the accumulated triplet concentration. As a result, *N*(*t*) in eqn (4) can be replaced by its stationary value, *N*(*t*) ≈ *N*_0_, which is derived from the following equation:^31^6
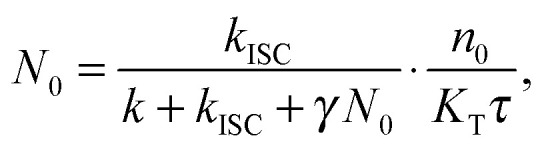
here *τ* is the time interval between two subsequent excitation pulses and 
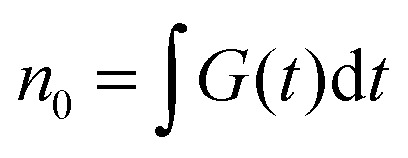
 is the total initial concentration of singlet excitons generated by a single pump pulse. The concentration of singlets decreases therefore faster in the annihilation regime than in annihilation-free conditions, but still in a simple single-exponential way:

7 *n*(*t*) = *n*_0_e^−(*k*+*k*_ISC_+*γN*_0_)*t*^.

Such a single-exponential behavior is indeed observed in, *e.g.*, polymer films, where a large concentration of singlet and/or triplet excitons is possible.^31,47^ Moreover, it was found that the triplet concentration in these polymer films can be up to three orders of magnitude larger than that of singlet excitons, *i.e.* there are multiple triplets present in one system at the same time. However, the maximum number of excitons in small photosynthetic antenna units like single LHCII complexes is limited to the number of available pigments. This implies that there is only a small number of triplets, up to one or two, present at the same time and that the simple kinetic model outlined above might be violated. This effect was indeed observed in our fluorescence measurements of single LHCII trimers that clearly demonstrated the appearance of two-exponential decay kinetics with relative amplitudes that strongly depend on the pumping intensity (Fig. 1). Therefore, to account for the limited amount of available exciton states and their discrete nature, a more detailed stochastic model has to be developed.

In small aggregates of pigment molecules like single LHCII trimers, distances between the most-remote chromophores are usually much smaller than the actual excitation diffusion length. As a result, the whole aggregate can be viewed as a single supermolecule which is fully characterized by a manifold of various accessible energy levels reflecting single and multiple excitations.^26,27,48,49^ The resulting stochastic model describing possible transitions between these energy levels has been successfully used to describe non-linear S–S annihilation in LHCII trimers.^32,33^ At a high repetition rate of the excitation laser, the formation of triplet states should also be considered, which requires the extension of the stochastic model of an LHCII supermolecule.

**Fig. 5:**
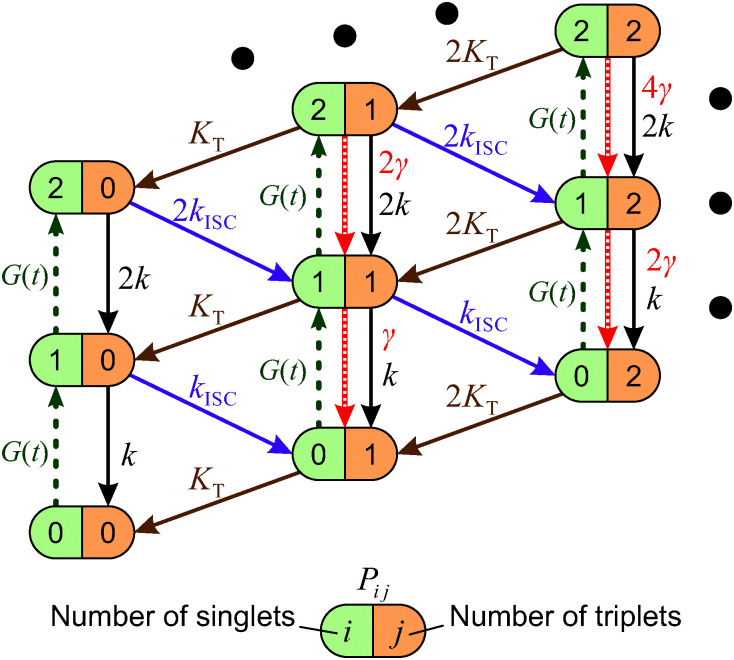
Stochastic model of S–T annihilation. Color ovals represent various possible states of the system containing different numbers of singlets and triplets. The probability of each state is *P*_*ij*_ and arrows demonstrate possible transition between these states. *k* and *K*_T_ are the relaxation rates of singlet and triplet states, respectively; *k*_ISC_ is the rate of inter-system crossing; *γ* is the rate of S–T annihilation; and *G*(*t*) denotes the generation rate of singlet states. The black dots indicate that the model can be farther extended to higher numbers of singlet and triplet states.

When the formation of triplet states is taken into account, the overall state of the system is fully described by two numbers, *i*—the actual number of singlets, and *j*—the actual number of triplets. If we denote the probability of this state as *P*_*i*,*j*_, the transitions between various states obey the following Pauli Master equations (see Fig. 5 for illustration):8
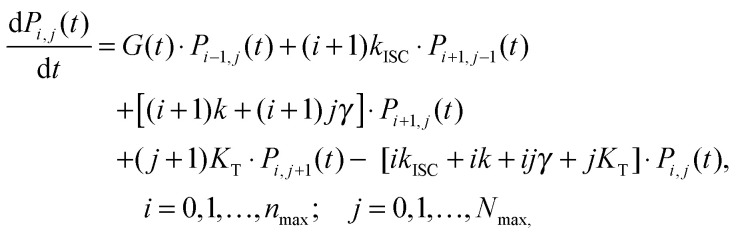
here all the rates are denoted in the same way as in eqn (4) and (5), whereas *n*_max_ and *N*_max_ represent the maximum number of the available singlet and triplet states, respectively. The numerical pre-factors of the transition rates in eqn (8) reflect the statistical number of possible relaxation pathways contributing to a particular transition in the supermolecule. The system of eqn (8) should be modified slightly at the boundaries of the network depicted in Fig. 5 in order to account for the lack of some transitions if *i* = 0 or *n*_max_ and *j* = 0 or *N*_max_. Since excitation intensities used in our experiments were rather low, the states corresponding to *i* ≥ 2 are expected to remain almost unpopulated. Therefore, for the sake of simplicity we neglect terms describing S–S annihilation in eqn (8) and Fig. 5; however, the model can be easily adjusted to account for this additional relaxation channel that becomes available at higher excitation densities, see *e.g.*ref. 33,50.

By numerically solving this system of differential equations, the time-dependent probabilities *P*_*i*,*j*_(*t*) of every state can be easily obtained. Then the mean number of singlets, *n*(*t*), is defined as a weighted sum:9
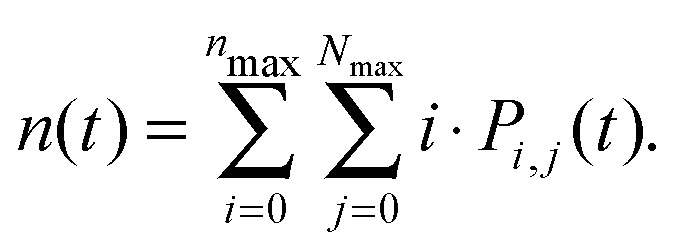


Analogically, the mean number of triplets is10
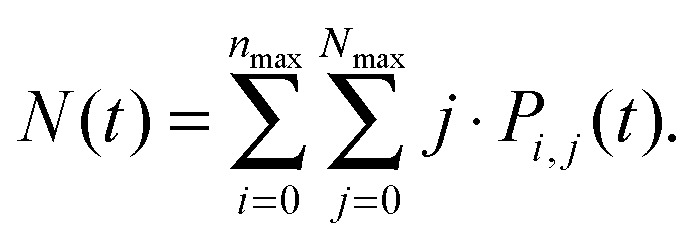
 If we analytically calculate the sums in eqn (9) and (10) by taking the Pauli Master equations (eqn (8)) into account, we obtain two simple relations:
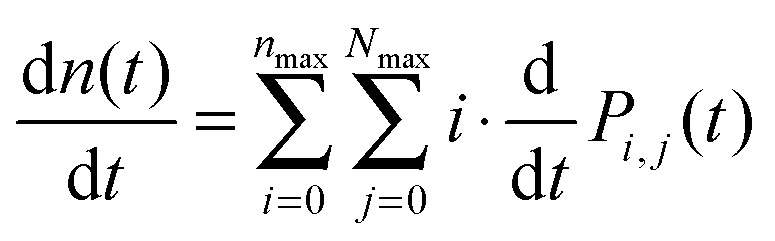
11
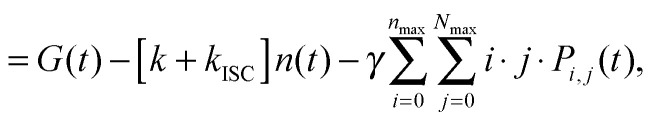
12
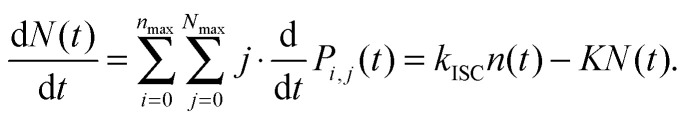
 These equations are exactly the same as eqn (4) and (5), except for the last term in eqn (11). This term is in fact the reason for the deviation from mono-exponential decay kinetics of singlet excitons. For short excitation pulses (compared to other characteristic time scales), the exact form of the generating function *G*(*t*) is not important—the only significant quantity is the initial population of singlets, 
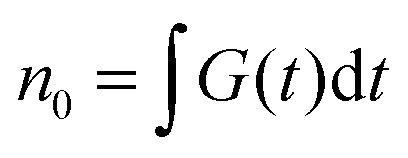
. Starting from the initial distribution *P*_0,0_(*t*) = 1 and *P*_*i*,*j*_(*t*) = 0 for *i* > 0 or *j* > 0, the system of eqn (8) can be solved for a large sequence of laser excitations until the quasi-stationary distribution of triplets is obtained, *i.e.* until the probabilities *P*_*i*,*j*_(*t*) prior to two subsequent pulses become indistinguishable.

The same model can also be used to simulate AOM histograms following some particular off-time period as discussed above. Indeed, the number of detected photons during a specific time bin interval Δ*t* at the AOM delay time *t*_AOM_ is proportional to the integral of the singlet kinetics:13
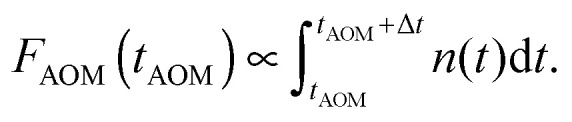
 For simplicity, if the binning time interval Δ*t* in eqn (13) is small compared to the timescale of formation of the triplet state, it can be substituted with the time interval *τ* between two subsequent excitation pulses while the proportionality in eqn (13) is still approximately preserved.

## 5 Modeling results

As discussed above, the average fluorescence decay kinetics from single LHCII trimers exhibit a two-exponential decay with a fast lifetime of *τ*_fast_ ≈ 35 ps and a slow value of *τ*_slow_ ≈ 3.4 ns. In terms of the stochastic description this indicates that on average less than one triplet state per LHCII is formed: the fast kinetics represents the case when exactly one triplet is generated, so that the corresponding lifetime is *τ*_fas*t*_ ≈ (*k* + *k*_ISC_ + *γ*·1)^−1^ ≈ *γ*^−1^. The slow kinetics, on the other hand, can be attributed to the case when no triplets at all are generated, yielding *τ*_slow_ ≈ (*k* + *k*_ISC_)^−1^. From these kinetics only the S–T annihilation rate *γ* ≈ 1/(35 ps) can be evaluated precisely, whereas all the other transition rates present in eqn (8) remain uncertain. It can be shown that various sets of the parameters *k*, *K*_T_, *k*_ICS_, and *n*_0_ can equally well reproduce the experimentally-observed fluorescence kinetics. To avoid ambiguity, it is necessary to obtain additional information on the rate of triplet formation.

This additional information is provided by the time-dependent AOM experiments illustrated in Fig. 3, revealing the process of triplet generation. To verify the proposed stochastic model of S–T annihilation, we used eqn (8) and (13) to simultaneously fit all four AOM histograms shown in the inset of Fig. 4 just by using different AOM off-time periods *t*_off_. In order to avoid any possibly remaining uncertainty in the fitting results, we also used slow and fast lifetimes extracted from the steady state fluorescence kinetics as additional constrains for the model parameters. Other variables like *t*_on_ = 5 μs, *τ* = 1/*f* = 13.16 ns (here *f* = 76 MHz is the laser repetition rate) and the excitation intensity *I*_E_ = 500 W cm^−2^ were fixed to represent the experimental conditions.

**Fig. 6:**
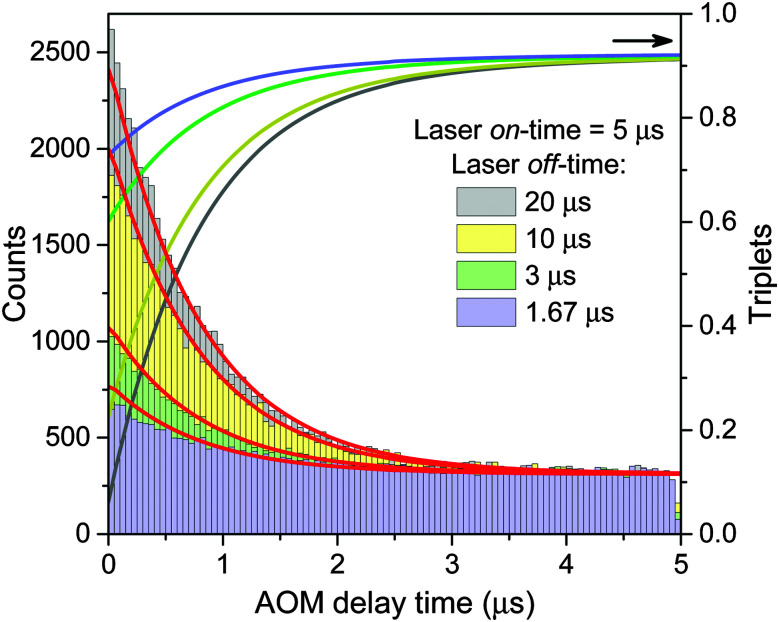
AOM histograms from the inset of Fig. 4. Red lines indicate best-fitted re-normalized values of the integral of singlet kinetics between two subsequent laser pulses, calculated at a given AOM delay time according to eqn (13) and using the parameters listed in Table 1. The calculated time evolution of the triplet states is shown with lines of the same color as the corresponding AOM histograms (right axis).

The obtained model parameters are outlined in Table 1 while the corresponding best-fitting AOM histograms are shown with red lines in Fig. 6. In the same figure we show the calculated rise kinetics of the triplet population. As expected and qualitatively described above, a higher amplitude of the AOM kinetics at the onset of a modulation cycle corresponds to a lower initial average concentration of triplets and thus a slower decay of singlet states of Chl molecules. To further validate the proposed model, we have used the same parameters listed in Table 1 to calculate two more AOM histograms corresponding to different modulation frequencies, duty cycles, and excitation intensities. The theoretical predictions are compared with the experimental results in Fig. S5 in the ESI† and show good agreement.

**Table 1:** Model parameters used to fit the AOM histograms in Fig. 6

Model parameter	Value^a^
S–T annihilation rate	*γ* ^−1^ = (36 ± 1) ps
Singlet linear relaxation rate	*k* ^−1^ = (5.81 ± 0.05) ns
Triplet linear relaxation rate	*K*_T_^−1^ = (6.99 ± 0.15) μs
Inter-system crossing rate^b^	*k*_ISC_^−1^ = (8.54 ± 0.03) ns
Initial excitation per 1 kW cm^−2^ of laser intensity	*n*_0_ = (0.073 ± 0.002)/1 kW cm^−2^

a Error estimates correspond to the 95% confidence interval.

b This rate includes inter-system crossing of Chls and subsequent triplet transfer to Cars.

**Fig. 7:**
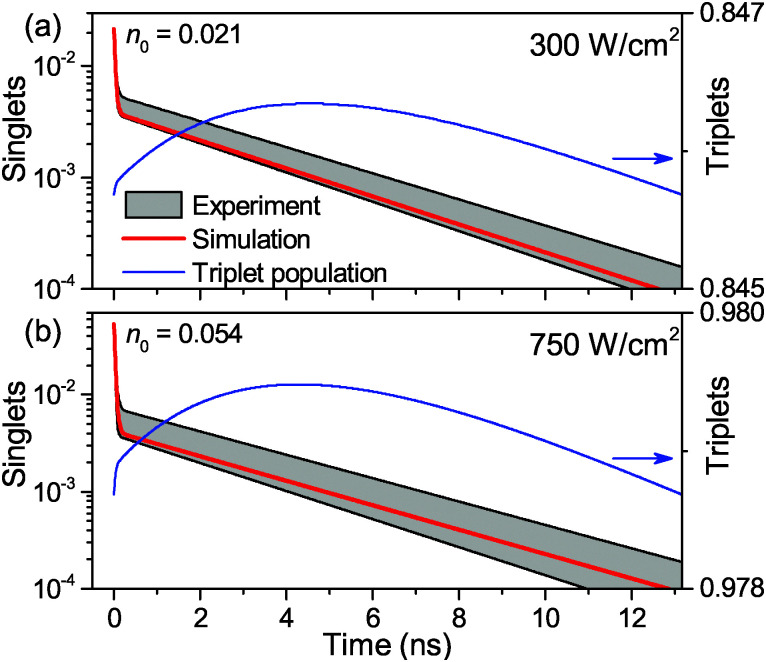
Calculated steady-state fluorescence kinetics of singlets (blue line, left axis) and triplets (red line, right axis) in single LHCII complexes for excitation intensities of 300 W cm^−2^ (a) and 750 W cm^−2^(b). *n*_0_ denotes the initial number of generated singlets and the gray shading indicates the boundary values of the standard deviation from the experimental measurements.

The calculated fluorescence decay kinetics, corresponding to the stationary population of the triplet states, indeed exhibit two-exponential behavior, as demonstrated in Fig. 7 for two different excitation intensities of 300 and 750 W cm^−2^. In both cases the concentration of triplets almost does not change between two subsequent laser pulses and is indeed smaller than 1 (on average 0.85 and 0.98 per LHCII trimer, respectively), as discussed above. As a result, the total singlet excitation kinetics are the statistical average of all possible triplet numbers: At an excitation intensity of 300 W cm^−2^ there is, for example, a 1.9% probability for the system to contain two triplets, a 80.7% probability for one triplet, and a 17.4% probability for no triplets. The probability for two triplets is almost negligible (and it is even smaller at lower excitation intensities) and cannot be resolved in the experimental measurements. In fact, by slightly changing the lifetimes in the exponents as well as their relative amplitudes, the calculated kinetics can be perfectly fitted with a two-exponential decay. As we see from Fig. 7, the relative amplitudes of the fast and slow components strongly depend on the excitation intensity. This dependence was further investigated and the results fully agreed with the experimental measurements, as illustrated by the red line in Fig. 2. It shows the dependence of the relative amplitude of the fast decay component on the initial excitation *n*_0_, calculated by using parameters listed in Table 1.

## 6 Discussion

The first finding from the time resolved data on single immobilized LHCII complexes is that their fluorescence lifetime in the unquenched state, *τ*_slow_ ≈ 3.5 ns, is the same as the long lifetime component of solubilized complexes. That implies that neither surface attachment nor any other SMS-related measuring condition (*e.g.*, large detergent-to-protein ratio) systematically affects any of the radiative and non-radiative decay rates. In fact, the contribution of a small fraction of intermediately quenched complexes, typically less than 20% depending on the sample batch,^34,41^ could also explain the higher number of up to three decay components needed to fit ensemble measurements.^51,52^ The fluorescence decay of both unquenched and intermediately quenched intensity levels, observed at low excitation intensities (when the fast 35 ps component can be neglected) was always mono-exponential with the decay rate being

14 *k*_slow_ = *τ*_slow_^−1^ = *k* + *k*_ISC_ + *k*_q_,

here *k*, *k*_ISC_ and *k*_q_ are the rates for singlet decay, inter-system crossing and quenching, respectively. The latter one accounts for the faster decay in intermediately quenched states. Averaging over the whole ensemble of LHCII trimers in solution results in the set of multiple decay components needed to reproduce the observed fluorescence kinetics. Thus implementing the technique of single molecule spectroscopy allowed us to disentangle quenching and/or bleaching effects and thus to focus solely on the properties of individual highly fluorescent unquenched LHCII trimers.

The main result of our work is the observation of a second fast lifetime component of ∼35 ps, appearing at excitation intensities exceeding 50 W cm^−2^. The relative amplitude of this fast component was found to depend heavily on the excitation intensity and saturated at 
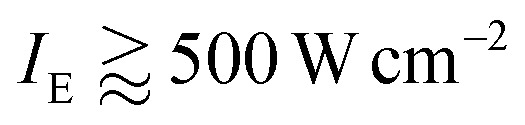
. Additional measurements, performed by utilizing an acousto-optic modulator and accompanied with numerical simulations, allowed us to unambiguously assign that fast decay component to S–T annihilation. On the other hand, the probability of S–S annihilation even for the highest excitation intensity of 1500 W cm^−2^, resulting in the absorption rate of roughly 1 photon per 10 pulses per LHCII trimer, is just about 0.5% and can therefore be neglected. Nevertheless, as was already mentioned, the proposed stochastic model can be straightforwardly extended to even higher pumping intensities by introducing additional relaxation channels accounting for S–S annihilation.

The fact that the two observed lifetime components can be distinguished in a single connected and equilibrated pigment–protein complex implies that they arise from mainly two distinct states of the complex. The presence of one (or possibly more) Car triplet states leads to the subsequent S–T annihilation events and therefore results in the fast decay component. Meanwhile, the slow component is the overall singlet excitation decay rate observed in the absence of any triplet state. The observed two-exponential decay is therefore a time-integrated sum of the stochastic interchange of both scenarios. The annihilation rate of *γ*^−1^ ≈ 36 ps contains information about the inter-pigment energy transfer processes and can be understood as the mean diffusion time of a singlet excitation until its energy is transferred to a Car triplet state and annihilated. It approximately corresponds to the so-called excitation equilibration time. Furthermore, this defined time constant for annihilation in an LHCII trimer implies a reasonably well-connected and structurally unchanged trimeric structure of the immobilized protein complex. Nevertheless, the width of the distribution of the fast lifetime might actually contain more information about the underlying energy transfer kinetics. Different energy transfer pathways within an LHCII trimer lead to an inter-pigment transfer rate distribution of hundreds of femtoseconds to tens of picoseconds. This indicates a strong fractal-like character of the annihilation rate^53^ and a broadening effect on the observed annihilation rate distribution at room temperature, in contrast to the light-harvesting antenna of the photosynthetic bacteria.^54^ One example of such a structural inhomogeneity is mutual location of the singlet and triplet states within the LHCII trimer: the singlet excitation can be located either within the same monomeric subunit as an existing triplet or in another one. In the later case, S–T annihilation is preceded by the inter-monomer excitation energy transfer. Static-disorder-induced differences in connectivity might also contribute to the width of the distribution, but unfortunately all these contributions are not easily distinguished from slight fitting uncertainties.

Another outcome is the successful application of a stochastic model to qualitatively and quantitatively describe the S–T annihilation kinetics. The proposed model was able to reproduce the two-exponential fluorescence decay as well as the excitation intensity dependence of the relative amplitude fractions of steady-state experiments. This redistribution of relative amplitudes explains the saturation behavior of the detected fluorescence intensity *I*_F_ which can be calculated as 
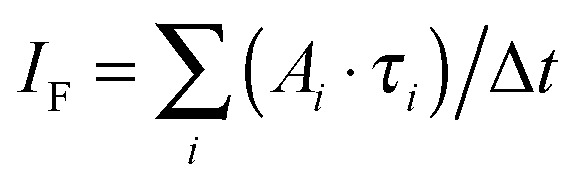
. At low excitation intensities the contribution of *A*_fast_ can be neglected, and the fluorescence intensity *I*_F_ scales approximately linearly with the amplitude *A*_slow_ and therefore the total excitation intensity. However, at higher excitation intensities the impact of the fast component increases and eventually starts to dominate the excitation decay kinetics, resulting in a saturation of the fluorescence intensity.

Time-dependent measurements of Car triplet generation and the Car triplet decay rate allowed us to further test and validate the model. The simultaneous fit of all the described experiments resulted in a set of parameters listed in Table 1. The slight deviations of fitted curves with the experimental data could have various reasons. The AOM decay kinetics shown in Fig. 3 are obtained from different single complexes and might thus indicate the influence of static disorder or structural heterogeneity. Further automation of the experiments to perform all measurements on one single complex might yield insights into that. Differences in Fig. 2 and 7 could meanwhile arise from the presence of an additional quenching mechanism that on average slightly decreases the probability of S–T annihilation. Fast blinking events that cannot be resolved in fluorescence intensity traces might be an explanation.^41^ These could be caused by conformational changes of the pigment–protein complex, but the reported presence of a low number of unquenched Chl triplets^15,55^ could also contribute, especially at higher excitation intensities.

The obtained initial excitation *n*_0_ (1 kW cm^−2^) = 0.073 represents the number of absorbed photons per laser pulse, which agrees well with the evaluated absorption rate of ∼0.06 photons per pulse based on the given excitation intensity and the reported absorption cross-section of an LHCII trimer of *σ* = 1.4 × 10^−15^ cm^2^.^38^ The experimentally obtained Car triplet decay rate of *K* ≈ (6.6 μs)^−1^ in anaerobic conditions is only slightly faster than the values of 7–9 μs found in literature,^14,46^ and the fitted value is even closer. This discrepancy might be caused by trace amounts of oxygen; however, that seems unlikely due to the high photo-stability of complexes (typically more than one minute). Another possibility is that S–T annihilation intrinsically shortens the lifetime of Car triplet states *via* the frequent access of higher excited triplet states. Meanwhile, the obtained inter-system crossing rate of 8.54 ns^−1^ agrees with the published range of ∼10 ns^−1 ^ and results in an absolute triplet yield of 40%.^14,20^ This is somewhat higher than the value of 30% found for PSII with closed reaction centers in chloroplasts.^9^ However, this discrepancy can be explained by the difference in the slightly quenched long lifetime component of about ∼2 ns in the latter case. The obtained results on the S–T annihilation kinetics for the given excitation rates are also approximately valid for continuous wave (CW) excitation as the time scale of the triplet decay is two orders of magnitude slower compared to the laser repetition rate utilized for this study. This includes the assumption that the mean photon absorption rate at a given average excitation intensity is the same for pulsed and CW excitation.

## 7 Conclusions

We present a quantitative and conclusive study on the process of S–T annihilation in small pigment–protein complexes, based on single molecule measurements of the antenna complex LHCII. The development and application of a statistical modeling approach enabled us to unambiguously assign the fast lifetime component of 35 ps to S–T annihilation. The experimentally observed two-exponential fluorescence decay can intuitively be understood as fast switching between an annihilation and a non-annihilation regime, corresponding to the presence and absence of a Car triplet state. Calculating the stochastic probability of triplet state generation and decay on the μs time scale allowed to fit all our experimental data and validate the proposed statistical model. The presented work therefore gives a detailed description of this intrinsic self-quenching mechanism in a single photosynthetic antenna complex. It will furthermore help to understand the S–T annihilation kinetics in molecular aggregates of various sizes and especially PSII supercomplexes that fall into the intermediate range between a stochastic and kinetic mathematical description.

## Supplementary Material

CP-017-C5CP01806D-s001
