# Supplementary material for: Singlet–triplet annihilation in single LHCII complexes
Source: Phys Chem Chem Phys. 2015 Jul 3;17(30):19844–53. doi: 10.1039/c5cp01806d (PMC11289710; doi:10.1039/c5cp01806d)
Supplement: CP-017-C5CP01806D-s001 [file CP-017-C5CP01806D-s001.pdf]

Electronic Supplementary Material (ESI) for Physical Chemistry Chemical Physics.
This journal is © the Owner Societies 2015

Singlet–Triplet Annihilation in Single LHCII Complexes

J. Michael Gruber,[a,*] **Jevgenij Chmeliov,**[b,c] **Tjaart P. J. Kruger,**[d] **Leonas Valkunas,**[b,c] **and Rienk van Grondelle**[a,*]

[a] Department of Biophysics, Faculty of Sciences, Vrije Universiteit, De Boeleaan 1081, 1081HV Amsterdam, The Netherlands
[b] Department of Theoretical Physics, Faculty of Physics, Vilnius University, Saulėtekio Ave. 9, LT-10222 Vilnius, Lithuania
[c] Institute of Physics, Center for Physical Sciences and Technology, Goštauto 11, LT-01108 Vilnius, Lithuania
[d] Department of Physics, Faculty of Natural and Agricultural Sciences, University of Pretoria, Private bag X20, Hatfield 0028, South Africa

*Corresponding authors: j.m.gruber@vu.nl, r.van.grondelle@vu.nl

Electronic Supplementary Information

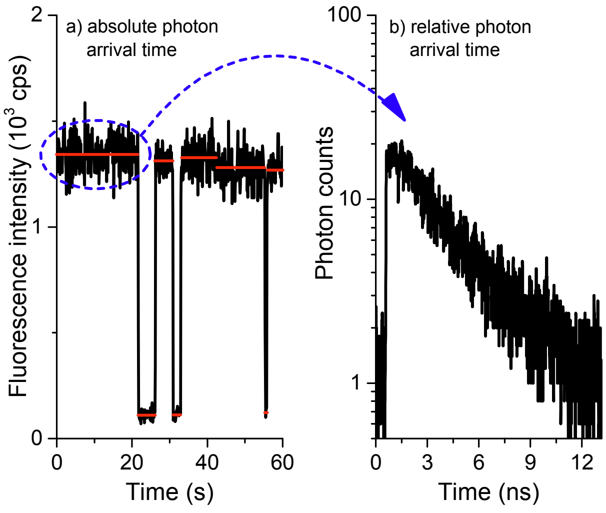

Fig. S1 Left side: illustration of a typical fluorescence intensity trace, reconstructed from the absolute arrival time of detected photons with a binning time of 10 ms (black line). The red lines indicate the fitted intensity levels used for further analysis. Right side: the fluorescence decay of the first intensity level, reconstructed from the relative arrival time of the detected photons.

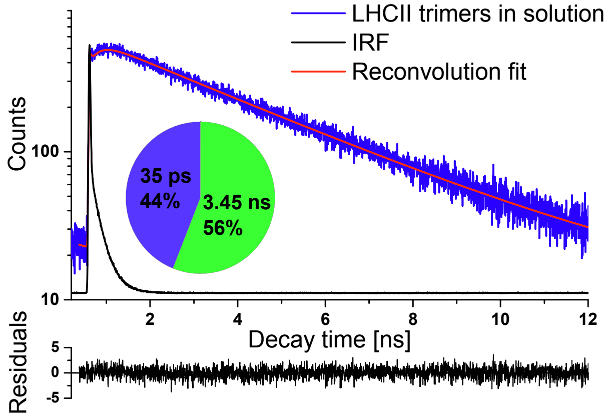

Fig. S2 Ensemble measurement of the fluorescence decay of solubilized LHCII trimers. Kinetics were measured at an average excitation power of 3 µW and can be fitted with similar lifetime components as kinetics obtained from the single-molecule measurements. The excitation intensity varies within the confocal excitation spot and, together with three-dimensional diffusion, this results in non-trivial annihilation kinetics that cannot be directly compared with the results of single complexes.

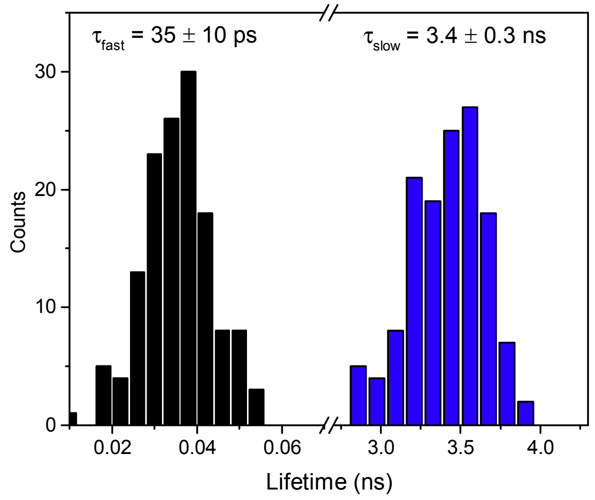

Fig. S3 Histogram of lifetimes from a two-exponential fit of 100 individually analyzed LHCII trimers.

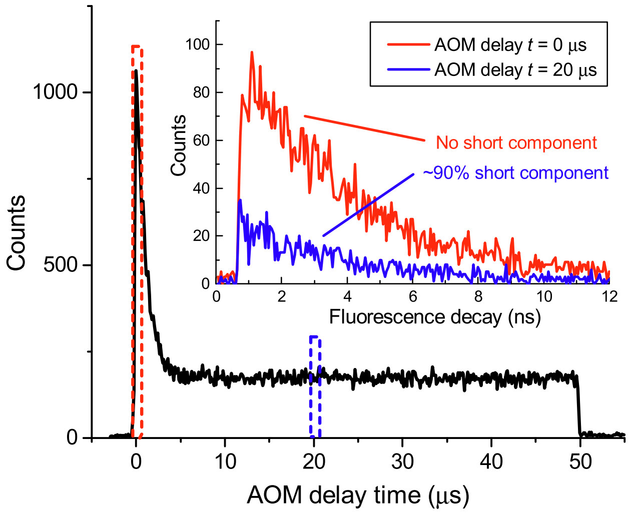

Fig. S4 AOM histogram, measured at $500\,\mathrm{W/cm^2}$ with *on*- and *off*-times of 50 µs. The inset shows the fluorescence decay (50-ps binning) of photons, detected in the first microsecond (red line) of the AOM modulation and during 1 µs at an AOM delay time of 20 µs (blue line).

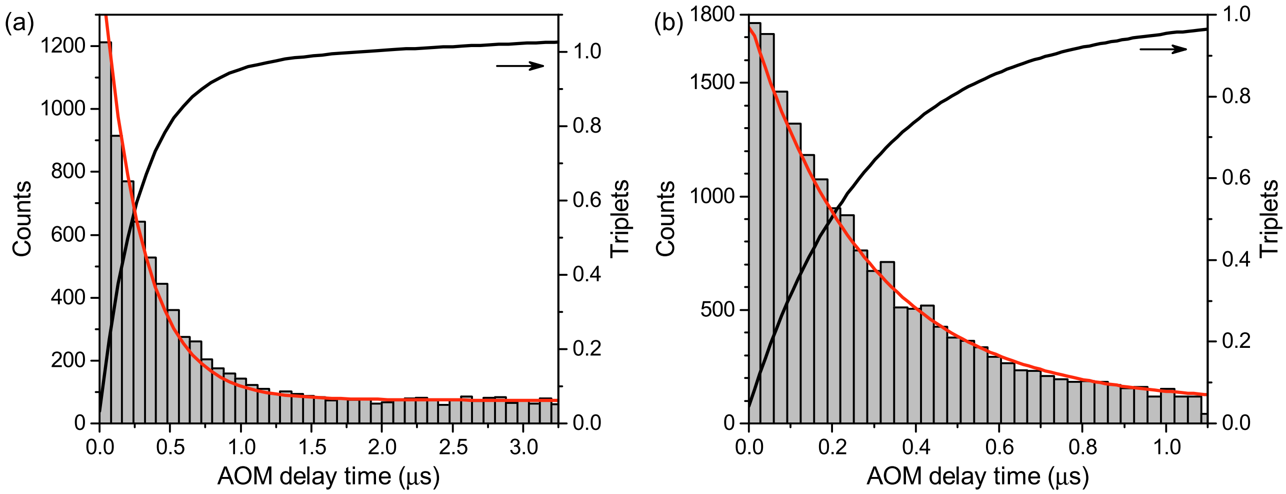

Fig. S5 AOM histograms, obtained at 10 kHz modulation frequency and 0.033 duty cycle yielding $t_{\mathrm{on}} = 3.3\,\mu\mathrm{s}$ (a) as well as at 30 kHz modulation frequency and 0.033 duty cycle yielding $t_{\mathrm{on}} = 1.1\,\mu\mathrm{s}$ (b). Excitation intensity was $1500\,\mathrm{W/cm^2}$ in both cases. Red lines indicate re-normalised values of the integral of singlet kinetics between two subsequent laser pulses, calculated at a given AOM delay time according to Eq. 13 using the parameters listed in Table 1 of the manuscript. The time evolution of the triplet states is shown with black lines (right axis).
